# Cultural values: can they explain self-reported health?

**DOI:** 10.1007/s11136-017-1512-x

**Published:** 2017-02-10

**Authors:** Bram Roudijk, Rogier Donders, Peep Stalmeier

**Affiliations:** Radboud University Medical Center, Radboud Institute for Health Sciences, Nijmegen, The Netherlands

**Keywords:** Self-reported health, Cultural values, World Values Survey, Multilevel modeling

## Abstract

**Purpose:**

Self-reported health (SRH) is a measure widely used in health research and population studies. Differences in SRH have been observed between countries and cultural values have been hypothesized to partly explain such differences. Cultural values can be operationalized by two cultural dimensions using the World Values Survey (WVS), namely the traditional/rational–secular and the survival/self-expression dimension. We investigate whether there is an association between the WVS cultural dimensions and SRH, both within and between countries.

**Methods:**

Data from 51 countries in the WVS is used and combined with macroeconomic data from the Worldbank database. The association between SRH and the WVS cultural dimensions is tested within each of the 51 countries and multilevel mixed models are used to test differences between these countries. Socio-demographic and macroeconomic variables are used to correct for non-cultural variables related to SRH.

**Results:**

Within countries, the survival/self-expression dimension was positively associated with SRH, while in most countries there was a negative association for the traditional/rational–secular dimension. Values range between 4 and 17% within countries. Further analyses show that the associations within countries and between countries are similar. Controlling for macroeconomic and socio-demographic factors did not change our results.

**Discussion:**

The WVS cultural dimensions predict SRH within and between countries. Contrary to our expectations, traditional/rational–secular values were negatively associated with SRH. As SRH is associated with cultural values between countries, cultural values could be considered when interpreting SRH between countries.

## Introduction

Self-reported health (SRH) is one of the most widely used health measures in academic research and is often included in population surveys, such as the European SHARE [1], the worldwide OECD PIAAC [2] studies, and the European Union Eurostat statistical bureau. It is used in demographic studies as a proxy for health or as an effective predictor for mortality [3, 4]. SRH has been studied extensively, but much remains unknown about the determinants of SRH. Several correlates have been proposed, mostly referring to respondents’ socio-demographic factors such as age, gender, education and social class [5–8]. Income and income inequality have been reported as important determinants for SRH [9, 10]. In this study, we consider culture as a determinant of SRH and use it to explain differences in SRH between countries.

Culture has been described as “The rich complex of meanings, beliefs, practices, symbols, norms and values prevalent among people in a society” [11]. Alternatively, as described by Hofstede et al. [12], culture consists of values and practices shared by a group. In general, values are related to norms wherein norms provide rules for behavior in specific situations, and values identify what should be judged as good or evil [13]. Alternatively, values have been defined as a set of stable, general beliefs that emerge from societal norms and individual psychological needs [14]. Scholars differ on how cultural values should be theorized. Schwartz has developed three cultural value dimensions, while other researchers, such as Hofstede et al. [12] and Rokeach [13] have developed theories on cultural values with even more cultural dimensions. Inglehart [15], Inglehart and Baker [16] has derived two cultural dimensions, using the World Values Survey (WVS). These dimensions, to be explained below, are labeled as “traditional versus rational/secular” and “survival versus self-expression”. Cultural values are known to differ between countries [17], which is illustrated by the Inglehart–Welzel cultural map [18, 19].

Having introduced cultural values, we consider their possible role in SRH within and between countries. Within countries, there is evidence that cultural values play a role in SRH. Zola has compared symptoms reported by Italian-American and Irish-American patients with an identical diagnosis [20, 21]. The Irish-Americans tended to attribute their complaints mainly to specific parts of the body such as the eye or ear, while expressing that they did not experience much pain. The Italian-Americans reported more vague complaints and stated that the complaints were interfering with their everyday lives, while also reporting more pain than the Irish-Americans. Summarizing, Zola showed that people from different cultures communicate differently about their health. A study by Diener et al. on well-being [22] is also relevant as well-being is related to SRH [23–25]. Diener states that people with characteristics valued within their culture tend to feel happier [22]. For example, they have found that self-esteem predicts well-being better in individualistic cultures than in collectivistic cultures [26]. These studies suggest that within countries, SRH may be influenced by cultural values.

Between countries, several studies have found evidence for a role of cultural values on SRH. Jürges found differences in mean SRH between countries and hypothesizes that cultural values may explain those differences [27]. Mackenbach [28] has studied the relation between health and cultural dimensions over European countries and found significant relations between these cultural dimensions and a variety of health behaviors, health outcomes and health policies. Diener et al. [29] report that well-being differs between cultures and offers different cultural standards for feeling and expressing positive emotions as a cause [22, 30]. These studies suggest that an association exists between cultural values and SRH, but now between countries.

The above findings give rise to the hypothesis that cultural values are related to SRH. The aim of this study is then to determine such a relation exists, both within and between countries. This leads to the following two research questions:

1. Is there an association between the WVS cultural dimensions and self-reported health within countries?
2. Is there an association between the WVS cultural dimensions and self-reported health between countries?

We formulate the following hypotheses. First, findings within countries suggest cultural values determine how people perceive their health [20–22]. This could lead to differences in SRH. Therefore, we hypothesize an association between cultural values and SRH within countries. Second, based on the evidence of Jürges [27], Mackenbach [28] and Diener [29], we hypothesize that there is an association between cultural values and SRH between countries. Third, as wealthier countries, with more sophisticated health care, tend to have positive scores on the two WVS cultural dimensions, we hypothesize that the WVS cultural dimensions are positively associated to SRH.

## Methods

### Rationale

The association found between countries, the ecological level, may not be representative for the associations within countries, the individual level, which troubles the interpretation of associations between countries. For instance, a positive association for cultural values and SRH may exist between countries, while a negative association exists within countries. Extrapolating the between country level to the individual level would then lead to a false inference. This problem is called the ecological fallacy. To avoid it, we assess the associations both between and within countries. Thus between country associations can be interpreted in the light of within country associations. Socio-demographic factors will be used as control variables, as their importance for SRH has been shown in previous studies. Macroeconomic variables are also included as control variables, as they can account for non-cultural differences between countries and are shown to be correlated with happiness [31].

### Measures

To fulfill the aims of this research paper, cultural values need to be operationalized into a quantifiable concept that discriminates between countries. The World Values Survey (WVS) Association has done so and their data is used here. The World Values Survey Longitudal Data [32] and the European Values Survey Longitudinal Data (EVS) [33] were merged to create the Integrated Values Survey (IVS) database, using the protocol provided by the WVS [34]. This dataset includes almost 100 countries, up to 6 waves per country, containing at least 1000 respondents per country and wave in most cases. The dataset contains 506,268 unique respondents and respondents only participate in one single wave. The sampling scheme is representative in each country [35]. The survey includes composite cultural values, socio-demographic variables and SRH. The IVS allows for a computation of two composite cultural dimensions: survival versus self-expression and traditional versus rational/secular.

**Fig. 1:**
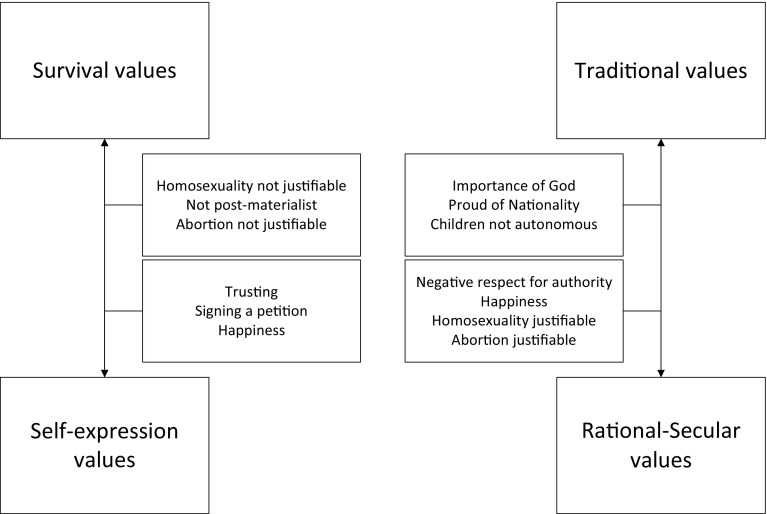
The cultural dimensions and their factor items

Figure 1 shows how the two composite cultural dimensions are described, based on factor analysis of 10 items. In the upper left of Fig. 1, survival values emphasize economic and physical security and have low levels of trust and tolerance. In the lower left, self-expression values correspond with higher levels of trust, tolerance and political activism. The upper right shows that traditional values are related to religion, authority, national pride, and parent–child ties. The lower right of the table shows rational–secular values, which are the opposite of traditional values. The two cultural variables are continuous. Negative scores on the traditional/rational–secular variable indicate that respondents have traditional values, while a positive score indicate that respondents have rational–secular values. Negative scores on the survival/self-expression dimension indicate that respondent have more survival values, while positive scores indicate self-expression values.

WVS researchers Ronald Inglehart and Christian Welzel have been able to create the Inglehart–Welzel cultural map [18, 19], which illustrates that countries can be differentiated by cultural values. European countries and English speaking countries score high on both self-expression and rational/secular values, while African and Islamic countries score low on these values. Asian and former Soviet countries score high on rational/secular, but lower on self-expression. Lastly, Latin American countries score high on self-expression and lower on secular/rational values.

The WVS and EVS databases contain a variety of socio-demographic variables, such as gender, education, income, self-perceived social class and age. Gender is coded as 0 for female and 1 for male. Education, income (position in the scale of incomes from lowest to highest) and self-reported social class are categorical variables that have 1 as lowest value and 5 or 10 as highest values. Self-reported health was a categorical variable coded as (1) Very good, (2) Good, (3) Fair, (4) Poor or (5) Very poor. SRH was reversed to (5) very good health to (1) very poor health. Macroeconomic data is obtained from the World Bank [36]. The macroeconomic variables are country level variables and include GDP per capita (Gross Domestic Product, a measure of wealth within a country, PPP 2011 US dollars), government health expenditure as percentage of the GDP, life expectancy at birth, total health expenditure per capita (PPP, 2011 US dollars) and out of pocket expenditure on health (as percentage of total spending on health).

### Analyses

For research question 1, information is needed on the coefficients of the association between cultural values and SRH, which will be provided by models 1 and 2. Regressions are performed for each country separately. In both models, SRH is the dependent variable and the two cultural dimensions are the independent variables. Model 2 also includes socio-demographic variables.

1 $$\text{SR}{{\text{H}}_{i}}={{\beta }_{i0}}+{{\beta }_{i1}}\text{Tradrat}+~{{\beta }_{i2}}\,\text{Survself}+\varepsilon$$

2 $$\text{SR}{{\text{H}}_{i}}={{\beta }_{i0}}+{{\beta }_{i1}}\text{Tradrat}+~{{\beta }_{i2}}\,\text{Survself}+{{\overline{\beta }}_{i3}}\overline{\text{Socio}}+\varepsilon ~$$

A subscript $i$ indicates a country, while variables and parameters with bars on top indicate that the parameter is a vector of control variables and their $\beta$’s. ${{\overline{\beta }}_{i3}}\overline{\text{Socio}}$ contains for example, the variables age, gender, scale of incomes, social class (subjective) and education, each with their own slope ${{\overline{\beta }}_{i3}}$ for each of the $i$ countries. Abbreviations are used to indicate the two cultural dimensions and the random error term is denoted by $\varepsilon$.

To test whether the intercepts and slopes from model 1 and 2 differ between countries, models 3, 4 and 5 are constructed. These multilevel mixed effects models include random slopes for the cultural variables and random country dependent intercepts. SRH is the dependent variable, the WVS cultural dimensions are the independent variables and country is the level variable.

3 $$\text{SRH}=\left( {{\beta }_{0}}+{{\mu }_{0}} \right)+\left( {{\beta }_{1}}+{{\mu }_{1}} \right)\text{Tradrat}+\left( {{\beta }_{2}}+{{\mu }_{2}} \right)\text{Survself}+\varepsilon$$

4 $$\text{SRH}=\left( {{\beta }_{0}}+{{\mu }_{0}} \right)+\left( {{\beta }_{1}}+{{\mu }_{1}} \right)\text{Tradrat}+\left( {{\beta }_{2}}+{{\mu }_{2}} \right)\text{Survself}+\overline{{{\beta }_{3}}}~\overline{Socio}+\varepsilon \sqrt{2}$$

5 $$\text{SRH}=\left( {{\beta }_{0}}+{{\mu }_{0}} \right)+\left( {{\beta }_{1}}+{{\mu }_{1}} \right)\text{Tradrat}+\left( {{\beta }_{2}}+{{\mu }_{2}} \right)\text{Survself}+{{\overline{\beta }}_{3}}\,\overline{\text{Socio}}+{{\overline{\beta }}_{4}}~\overline{\text{Macro}}+~\varepsilon$$

The mixed models can be interpreted as following: $\left( {{\beta }_{0}}+{{\mu }_{0}} \right)$ is the intercept for each country, with a fixed part ${{\beta }_{0}}$ and a random part ${{\mu }_{0}}$, which allows for a constant intercept and a country dependent deviation of the intercept. $\left( {{\beta }_{1}}+{{\mu }_{1}} \right)$ and $\left( {{\beta }_{2}}+{{\mu }_{2}} \right)$ are the slopes for the cultural dimensions and consist of a fixed part ${{\beta }_{1}}$ or ${{\beta }_{2}}$ and a random, country dependent part ${{\mu }_{1}}$ or ${{\mu }_{2}}$, which allows again for a constant slope and a country dependent deviation of the slope. We assume that ${{\mu }_{0}}$, ${{\mu }_{1}}$ and ${{\mu }_{2}}$ are multivariate normally distributed with mean 0 and have an unstructured covariance matrix. If a random effect is significant, corresponding intercept or slopes differ reliably between countries. Again, socio-demographic variables are included in model 4. Model 5 adds macroeconomic control variables, to account for non-cultural differences between countries. Socio-demographic and macroeconomic variables are centered in all the models, creating a mean of 0 for all these variables.

For research question 2, information is needed about the association between countries. As multilevel models do not generate such an association between countries, a simple regression analysis with country level values for the cultural values and SRH was performed, to determine an association for the cultural dimensions and SRH between countries. Model 6 provides a mathematical representation of this regression analysis.

6 $$\text{SRH}=~{{\beta }_{0}}+{{\beta }_{1}}\text{Tradrat}+{{\beta }_{2}}\text{Survself}+{{\overline{\beta }}_{3}}\,\overline{\text{Socio}}+\varepsilon$$

As in the earlier models, ${{\overline{\beta }}_{3}}\,\overline{\text{Socio}}$ is a vector of socio-demographic variables and their slopes.

## Results

A sample from the IVS was obtained, including 506,268 respondents. Some of the macroeconomic data was not available for the first two waves of the WVS, which led to the exclusion of the first two waves. The unavailable macroeconomic data concerns mostly health-related variables, while variables such as GDP per capita were available and led to the exclusion of 92,456 cases. Furthermore, not all questions were asked in each country and wave, which led to the exclusion of 142,468 more cases. Missing macroeconomic data and unasked questions reduced the dataset to 271,344 cases. An additional 113,761 cases contained missing values for SRH, the cultural values or socio-demographic data, leaving 157,583 cases from 51 countries to be used for our within-country analyses. The mixed models contained a minimum of 45 countries, totaling 100,590 respondents. The difference between the sample sizes of the regression models and the mixed models is caused by missing macroeconomic data, mainly in wave 3 of the WVS. No data estimation for missing data was used.

**Fig. 2:**
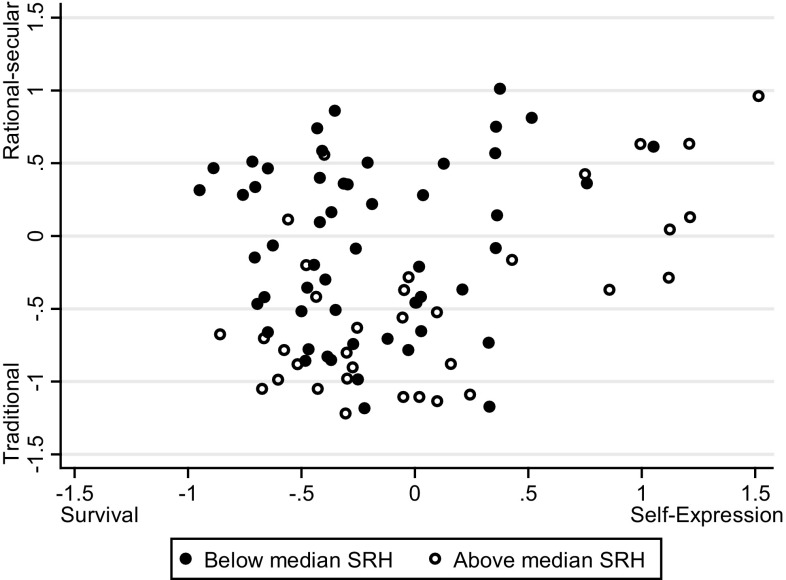
Cultural map, by mean SRH

Means of SRH differ per country; all countries in the sample have a mean between 3.1 and 4.4. Figure 2 illustrates that countries can be mapped into a two-dimensional plane, based on their scores on the two cultural dimensions and shows whether the country mean is below (black dot) or above (white dot) the median (3.85) of SRH.

**Fig. 3:**
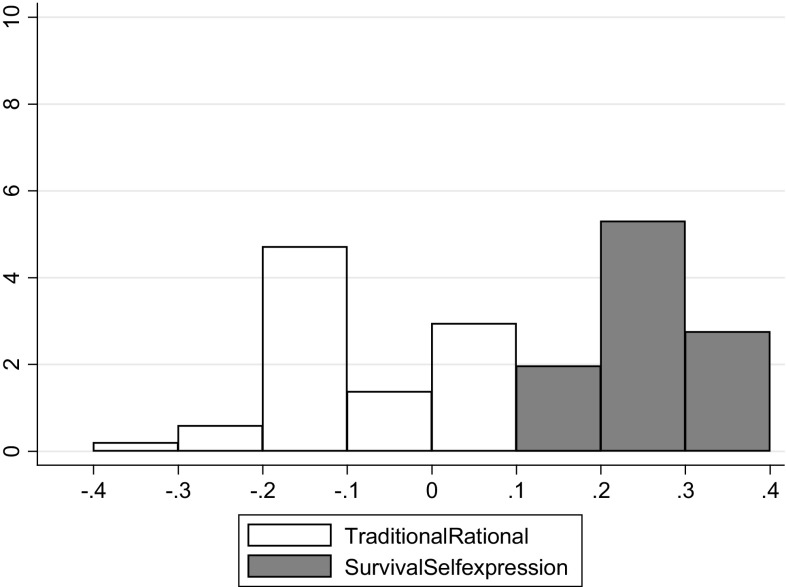
Regression coefficients per country for model 1

**Fig. 4:**
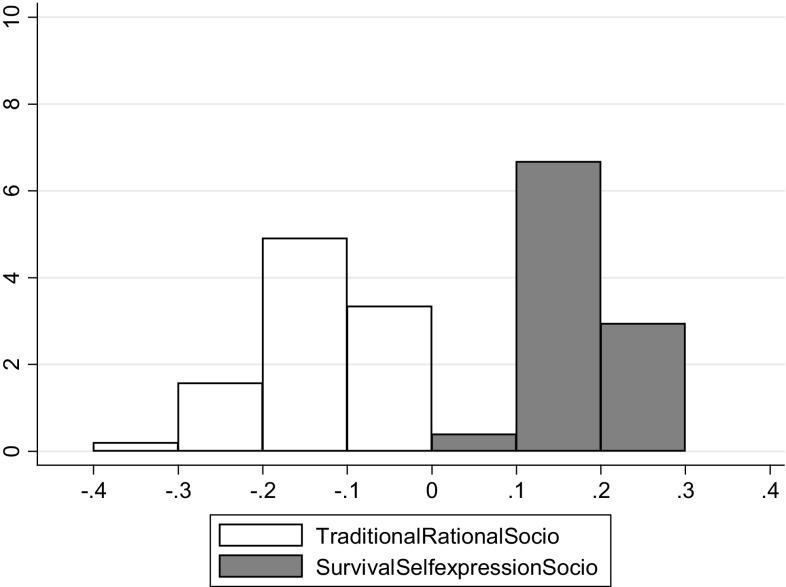
Regression coefficients per country for model 2, including socio-demographic variables

Regressions for SRH and cultural values were performed for 51 countries, using models 1 and 2. The coefficients for each cultural dimension per country are presented in histograms in Figs. 3 and 4. The coefficients for the traditional/rational–secular variable are represented by the white bars in the histograms, while the coefficients for the survival/self-expression variable are represented by the grey bars. Figure 3 shows the regressions without socio-demographic variables (averages are 0.258 for the survival/self-expression variable and − 0.089 for the traditional/rational–secular variable, while ${{R}^{2}}$ values ranged between 4 and 17% within each country), Fig. 4 shows the regression coefficients that are corrected for socio-demographic variables (averages are 0.178 for the survival/self-expression variable and − 0.140 for the traditional/rational–secular variable, ${{R}^{2}}$ values ranged between 6 and 37% within each country).

The results of the mixed models, Eqs. (3), (4) and (5), are presented in Table 1. Model 3 contained only cultural variables, while model 4 includes socio-demographic control variables and model 5 contained both socio-demographic and macroeconomic control variables. The upper part of the table shows the coefficients of the fixed effects for each model. These fixed effects represent the average slopes of the cultural variables and the average intercept within countries. Socio-demographic and macroeconomic control variables are also included. Random effects are presented in the lower part of the table and represent the variation from the fixed effect for that variable between countries, expressed as a standard deviation. There is again a significant association between cultural values and SRH, which can be seen from the coefficients for the fixed effects. For the traditional/rational–secular variable, there is a negative association with SRH, while there is a positive association for the survival/self-expression variable and SRH. The random effects for the cultural variables and the constant are significant as well, indicating that the slopes of these variables differ between countries.

**Table 1:** Mixed effects models with self-reported health as the dependent variable

	Model 3 $$N=157,583$$	Model 4 $$N=157,583$$	Model 5 $$N=100,590$$
Fixed effects			
Traditional/rational–secular	−0.071**	−0.122**	−0.140**
Survival/self-expression	0.265**	0.188**	0.188**
Age		−0.013**	0.013**
Education		0.020**	0.018**
Social class (subjective)		0.054**	0.052**
Income scale (subjective)		0.027**	0.027**
Gender		−0.091**	−0.080 **
GDP per capita (in 1000$)			0.00048**
Health expenditure per capita (in 1000$)			0.0046**
Life expectancy			0.019 **
Government health expenditure (% of GDP)			0.084**
Out of pocket health expenses			0.006**
Constant	3.844**	3.874**	3.856**
Random effects			
SD traditional/rational–secular	0.093**	0.074**	0.074**
SD survival/self-expression	0.062**	0.049**	0.038**
SD constant	0.215**	0.188**	0.266**

**$$p<0.01$$

The between country regression coefficients from model 6 are reported in Table 2. Only significant variables are included. The coefficients of this model are similar to those of the mixed models and there is a negative association for the traditional/rational–secular dimension and a positive association for the survival/self-expression dimension. The ${{R}^{2}}$ is 34%. The effects of the cultural variables on SRH between countries can be sizable. Combining the range of cultural values from Fig. 2 with the coefficients from Table 2, the between country effect of cultural values on SRH corresponds with a change of about 0.75 on a 5 point scale, that is around 15%.

**Table 2:** Model 6: between country regression with SRH as the dependent variable

*N*	51
*R* ^2^	0.3352
Traditional/rational–secular	−0.143*
Survival/self-expression	0.239**
Age	−0.022**
Income	0.214**
Constant	3.824**

**p < 0.01, *p < 0.05

## Discussion

Our main finding is that the associations between the two cultural dimensions and SRH are similar both within and between countries. Within countries, there is a positive association between the survival/self-expression dimension and SRH and a negative association between the traditional/rational–secular variable and SRH. The amount of variance explained by cultural values varied between 4 and 17% for each country. Adding socio-demographic and macroeconomic variables left the association between the two cultural dimensions and SRH unchanged, while doubling the amount of variance explained. Between countries, similar associations were found as within countries. Cultural values can result in a 0.75 change on the 5 point SRH scale in the extreme case. The associations within and between countries were similar, although the slopes and average SRH differ between countries.

### Ecological fallacy

Our results show a similar association between cultural values and SRH within and between countries. This similarity is relevant, as within and between country associations are not necessarily the same. It is possible that individual associations do not hold on a country level or vice versa. Our results present evidence that these associations are similar, which allows us to extrapolate the association between cultural values and SRH from one level to the other. Thus we avoid the ecological fallacy of making inferences at the ecological level, while the association at the individual level is unknown. This is an important result of our research, as we can now justify claims about the association between countries by similar findings at the individual level.

### Associations between cultural values and SRH

From our Western European point of view, we hypothesized that both cultural dimensions would be positively associated with SRH. Western countries score relatively high on rational–secular and self-expression values and are usually considered to have sophisticated health care. In agreement with this, the survival/self-expression variable was positively associated with SRH, confirming a finding by Inglehart and Baker [16]. This is plausible, as self-expression values are related to tolerance for abortion and homosexuality, happiness and trust. A more trusting environment, as shown by Mansyur et al [5], and happiness [37] could lead people to report better health. Furthermore, Inglehart [15] argues that countries, scoring high on self-expression, shift away from an emphasis on economic growth and security towards an emphasis on quality of life [28]. As a consequence, this could lead these countries to implement policies to improve the quality of life of the population.

Contrary to our hypothesis, the traditional/rational–secular dimension was negatively associated to SRH. This implies that traditional values are related to higher SRH and rational–secular values to lower SRH. We can only provide ad hoc explanations for this relation. Traditional values are related to a high importance of authority, intolerance for abortion and homosexuality, religion and family ties, while rational–secular values imply the opposite. It is well known that family ties and social support play a role in the well-being of individuals, shown by high correlates of social functioning and mental and physical health in quality of life measures [38]. This suggests that strong family ties and higher levels of social support could lead to a higher SRH. Alternatively, religion may play a role in the relation between traditional values and SRH. Religious communities may provide social support, which could have an effect on SRH. Furthermore, religion reduces health-risk behavior such as substance abuse [39, 40] and could therefore potentially lead to higher SRH. Religious coping could also play a role [41, 42]. For instance, positive religious coping such as surrendering, putting your fate in God’s hands, is positively associated with mental health and quality of life. However, negative religious coping, believing that your illness is a punishment from God, is negatively associated with physical health [41]. Taken together, it is unclear how religious coping affects SRH. Summarizing, several explanations have been put forward, but no firm conclusions can be drawn. Future research is needed to interpret the relation between traditional values and SRH.

Between countries, differences in SRH, and also differences in the associations between cultural values and SRH have been found. These differences, shown by the significant random effect in slopes in the mixed models, will not be further explored. A purely methodological explanation could be, for example, measurement error. Another explanation might be that cultural concepts or health differ between countries. The significant random intercepts in the mixed models show that SRH differs between countries, confirming earlier findings. The variance in slopes and intercepts suggests that SRH is not only explained by cultural values, socio-demographic and macroeconomic variables, but also by other unaccounted differences between countries.

### Limitations and strengths

One of the limitations of this study is that the IVS and Worldbank databases contain many missing values. The original IVS database included some 500,000 respondents, while our final dataset contains only 157,583 respondents for the within country regressions and 100,590 respondents for the mixed models. This reduction is mainly caused by missing macroeconomic data and unasked survey questions in a substantial amount of the waves in the IVS. This accounts for around 230,000 missing values, while around 110,000 missing values arise for other reasons. Additional analyses revealed no substantial differences between the final dataset and the data that was excluded from analysis and the remaining dataset is still very large and contains more than 1000 respondents per wave for each country in most cases. Therefore, we assume that there is no systematic selection bias. Another limitation is that we make the assumption that the association between cultural values and SRH does not change over time. WVS data were collected in waves, but these waves were collapsed into a single wave because our focus is on differences between countries and not on temporal effects. Furthermore, another limitation of our study is that cultural subgroups within countries may have a different relation between cultural values and SRH than the relation at the aggregate group level (that is country level), for which we cannot control. Furthermore, other literature suggests that differences in response styles might cause differences in SRH between countries [27]. However, in the WVS, no information on response styles is available, which is a limitation. In addition, we acknowledge the limitation of using linear regression analysis for the within-country analyses. The SRH variable is a categorical variable, for which a conditional probit model would have been more appropriate. However, using regression models simplifies the interpretation of the within and between country models, which is why we chose linear regression models.

The main strength of this study is that we avoid the ecological fallacy. Another strength of this study is that it confirms the associations between SRH and socio-demographic and macroeconomic variables found in earlier studies [5–9]. Lastly, a major strength of this research is the large sample size from the WVS.

## Conclusion

SRH is widely used in academic research and population studies as a measure of health. Large cross-country studies such as the Survey of Health, Aging and Retirement in Europe (SHARE) [1], the OECD Survey of Adult Skills (PIAAC) [2] and the European Union Eurostat bureau include SRH in their surveys to support policy analyses. The aim of this paper was to assess the association between cultural values and SRH within countries and between countries. We found that associations between the WVS cultural dimensions and SRH within countries and between countries are similar and this can lead to a change of up to 0.75 on the 5 point SRH scale. Contrary to our expectations, the traditional/rational–secular dimension was negatively associated to self-reported health. As SRH is associated with cultural values between countries, cultural values could be considered when interpreting SRH between countries.
